# Description of *Rotylenchus rhomboides* n. sp. and a Belgian population of *Rotylenchus buxophilus* (Tylenchomorpha: Hoplolaimidae)

**DOI:** 10.21307/jofnem-2019-023

**Published:** 2019-04-24

**Authors:** Huu Tien Nguyen, Quang Phap Trinh, Marjolein Couvreur, Phougeishangbam Rolish Singh, Wilfrida Decraemer, Wim Bert

**Affiliations:** 1 Institute of Ecology and Biological Resource, Vietnam Academy of Sciences and Technology, 18 Hoang Quoc Viet, Cau Giay, Hanoi, Vietnam; 2 Graduate University of Science and Technology, Vietnam Academy of Sciences and Technology, 18 Hoang Quoc Viet, Cau Giay, 100000 Hanoi, Vietnam; 3 Nematology Research Unit, Department of Biology, Ghent University, K.L. Ledeganckstraat 35, 9000 Ghent, Belgium

**Keywords:** 28S–D2D3, Hierarchical cluster, *COI*, *Dioscorea tokoro*, ITS, Morphology, *Musa basjoo*, Phylogeny, Plant-parasitic, *Rotylenchus*, Systematic, Taxonomy

## Abstract

During a survey in the Botanical garden of Ghent University, a new species *Rotylenchus rhomboides* n. sp. and a population of *Rotylenchus buxophilus* were found. *Rotylenchus rhomboides* n. sp. is characterized by the presence of a rhomboid-like widening of the mid-ridge of lateral field at the level of vulva, a feature previously unknown within the genus. The population of the new species, composed only by females, has a rounded labial region with 4 to 5 annuli, robust stylet (31–37 μm long), short dorsal esophageal gland (9–19 μm) overlap of the intestine, vulva located slightly posterior to mid-body, and hemispherical or rounded tail shape with large phasmids located 3 to 5 annuli anterior to the level of anus. The hierarchical cluster analysis based on morphological features indicated that the new species closely resembles *R. corsicus*, *R. gracilidens*, and *R. rugatocuticulatus*. The DNA analyses of the D2-D3 of 28S rDNA, ITS rDNA, and *COI* mtDNA sequences of *Rotylenchus rhomboides* n. sp. show a close relationship with *R. buxophilus*, *R. goodeyi*, *R. laurentinus*, *R. pumilus*, and *R. incultus*, all of which can also be differentiated from the new species by morphological features. The combination of morphological, morphometric, and molecular characteristics confirmed the new species and the first report of *R. buxophilus* on yam (*Dioscorea tokoro*) in Belgium.

The type species *Rotylenchus robustus* (De man, 1876) was originally described as *Tylenchus robustus* by De man (1876). In a publication concerning free-living nematodes and their relation to the parasitic nematodes, Filipjev (1934) proposed the new genus *Rotylenchus* based on the type species *T. robustus* (De man, 1876).

The genus *Rotylenchus* is widely distributed all over the world and has been recorded from all continents with 103 valid species to date (Castillo and Vovlas, 2005; Vovlas et al., 2008; Atighi et al., 2011; Cantalapiedra-Navarrete et al., 2012, 2013; Atighi et al., 2014; Aliramaji et al., 2015; Noruzi et al., 2015; Talezari et al., 2015; Golhasan et al., 2016). According to Castillo and Vovlas (2005), the number of *Rotylenchus* species is second highest in Europe, after Asia. Several species of *Rotylenchus* are of economic importance in agriculture, among them, only *R. robustus*, *R. buxophilus* (Golden, 1956), *R. uniformis* (Thorne, 1949; Loof and Oostenbrink, 1958), and *R. goodeyi* (Loof and Oostenbrink, 1958) have been reported in Belgium (Steel et al., 2014; Viaene et al., 2017). They are the main cause of yield losses in many agricultural crops such as carrot, olive, orange, mango, soybean, broccoli, cabbage, tomatoes, etc. *Rotylenchus robustus* is considered as the most common species worldwide and has been reported in 25 countries and islands on all continents except Antarctica. *Rotylenchus buxophilus* is also a widely distributed species, occurring in Europe, Asia, North America, and New Zealand. *Rotylenchus uniformis* was found in the Netherlands and New Zealand. *Rotylenchus goodeyi* was recorded from several localities in the UK, the Netherlands, Belgium, Bulgaria, Slovakia, Spain, Switzerland, Luxemburg, and Uzbekistan (Castillo and Vovlas, 2005).

Herein, the new species *Rotylenchus rhomboides* n. sp. is characterized and a population of *R. buxophilus* is reported for the first time on yam (*Dioscorea tokoro* Makino). Both species were described based on morphology and morphometrics along with molecular characteristics and phylogeny of the D2-D3 expansion segment of 28S rDNA, ITS rDNA, and *COI* mtDNA sequences.

## Materials and methods

### Sampling and nematode extraction

The soil and root samples were collected around the rhizosphere of banana (*Musa basjoo* Siebold & Zucc. ex Iinuma) (GPS coordinates N: 51°2′6.8″, E: 3°43′22.7″) and Yam (*Dioscorea tokoro*) (GPS coordinates: N: 51°2′6.9″, E: 3°43′22.6″) at the Botanical garden of Ghent University. The nematodes were extracted from soil and roots by the modified Baermann tray method (Whitehead and Hemming, 1965).

### Morphological characterization

Nematodes were fixed in Trump’s fixative (2% paraformaldehyde + 2.5% glutaraldehyde in a 0.1 M Sorenson buffer (Sodium phosphate buffer at pH 7.3)), then dehydrated for mounting in glycerin on permanent slides following the method described by Singh et al. (2018).

Microphotographs and drawings were made from permanent slides by using an Olympus BX51 DIC Microscope equipped with a drawing tube and digital camera. Measurements were obtained based on light microscopic pictures using the software ImageJ 1.51. Illustrations were made by Illustrator ® CS 3 based on pencil drawings and SEM pictures. For scanning electron microscopy, specimens were processed and viewed following the procedure of Steel et al. (2011).

### Molecular characterization

Digital light microscope pictures were taken from temporary slides as morphological vouchers. Then, the nematodes were cut into pieces and put in the Eppendorf tubes with 20 µl of WLB (50 mM KCl; 10 mM Tris pH 8.3; 2.5 mM MgCl2; 0.45% NP 40 (Tergitol Sigma); 0.45% Tween 20) and were frozen for at least 10 min at −20°C. One μl proteinase K (1.2 mg ml^−1^) was added before the incubation in a PCR machine for 1 hr at 65°C and 10 min at 95°C and centrifugation for 1 min at 14,000 rpm. Finally, the samples were stored at −20°C before running PCR (Singh et al., 2018).

The 5′-end of the 28S rDNA region was amplified using the primers DP391/501 (Nadler et al., 2006) with the PCR reaction started at 94°C for 4 min, followed by 5 cycles of 94°C for 30 s, 45°C for 30 s, and 72°C for 2 min. This step was followed by 35 cycles of 94°C for 30 s, 54°C for 30 s, and 72°C for 1 min and finished at 12°C for 10 min. For ITS rDNA region, the primers Vrain2F/Vrain2R (Vrain et al., 1992) were used with the PCR reaction started at 94°C for 4 min, followed by 50 cycles of 94°C for 30 s, 54°C for 30 s, and 72°C for 2 min. The cytochrome c oxidase subunit 1 (*COI* mtDNA) gene was amplified using the primers JB3/JB4 according to the protocol of Derycke et al. (2010). The successful PCR reactions were purified and sequenced commercially by Macrogen Europe (Amsterdam, Nederland).

The forward and backward sequences were assembled in Geneious R11 (www.geneious.com) to get the consensus sequences. All the contigs were used for the BLAST search on GenBank to check for the closely related species (Altschul et al., 1997). Multiple alignments of the different sequences of each gene were made using MUSCLE in MEGA 7 (Kumar et al., 2016). The phylogenetic trees were created by using MrBayes 3.2.6 (Huelsenbeck and Ronquist, 2001) Add-in in Geneious R11 (www.geneious.com) under the nucleotide substitution models that were selected by using MEGA 7 (Sharma et al., 2012) based on the BIC criterion. The selected models were HKY + G for the D2-D3 of 28S rDNA and ITS rDNA sequences, and GTR + G + I for *COI* mtDNA sequences. The Markov chains were set with 1 × 10^6^ generations, 4 runs, 20% burn-in, and subsampling frequency was 500 generations (Huelsenbeck and Ronquist, 2001). For D2-D3 of 28S rDNA data set, *Helicotylenchus dihystera* (accession number: AB933471) and *Helicotylenchus multicinctus* (accession number: MF401446) were chosen as outgroups. The outgroups for ITS rDNA data set were *Hoplolaimus columbus* (accession number: FJ485623) and *Hoplolaimus seinhorsti* (accession number: KX446971), and *Scutellonema brachyurus* (accession number: JX472089) and *Scutellonema truncatum* (accession number: KX959308) were selected for *COI* mtDNA data set.

### Cluster analysis and web-based key

The new species was compared with 103 described species, based on the tabular key of Castillo and Vovlas (2005). This was done using Hierarchical Cluster analysis implemented in the software Primer 6 (Clarke and Gorley, 2006) using Bray–Curtis similarity measure with the percent similarity between species defined by the average of the multiple characters. The 11 characters of 103 described species and *Rotylenchus rhomboides* n. sp. in the analysis are: (A) Lip annulation: 1: absent or smooth labial region, 2: labial region with 2 to 3 annuli, 3: labial region with four annuli, 4: labial region with five annuli, 5: labial region with six annuli, 6: labial region with 7 to 8 annuli, 7: labial region with 9 to 10 annuli; (B) Labial region shape: 1: labial region hemispherical, 2: labial region rounded, 3: labial region conoid, 4: labial region truncate; (C) Lateral field areolation: 1: areolated only in esophageal region, 2: areolated in esophageal region and irregularly areolated at mid-body, 3: areolated in esophageal region and incompletely areolated at mid-body, 4: areolated in esophageal region and near phasmids, 5: areolated along whole length of body except at tail region, 6: areolated along whole length of body including the tail region, 7: incompletely areolated along whole body; (D) Body longitudinal striations: 1: punctated along body annuli, 2: longitudinally striated in esophageal region, 3: longitudinally striated over whole body, 4: without body striations; (E) Stylet length: 1: stylet shorter than 30 µm, 2: stylet between 30 and 35.9 µm, 3: stylet between 36 and 40.9 µm, 4: stylet longer than 41 µm; (F) Dorsal esophageal gland outlet (DGO) to stylet base: 1: DGO less than 2 µm, 2: DGO between 2 and 6.9 µm, 3: DGO between 7 and 12 µm, 4: DGO > 12 µm; (G) Dorsal esophageal gland overlapping: 1: esophageal gland overlapping less than 5 µm, 2: esophageal gland overlapping by 6 to 20.9 µm, 3: esophageal gland overlapping by 21 to 30.9 µm, 4: esophageal gland overlapping by 31 to 40.9 µm, 5: esophageal gland overlapping by >41 µm; (H) Tail shape: 1: hemispherical, 2: rounded, 3: conoid, 4: pointed, 5: with ventral projection; (I) Ratio V: 1: ratio V < 50%, 2: ratio V of 50 to 70%, 3: ratio V > 70%; (J) Presence of males: 1: males present, 2: males absent; (K) Phasmid position: 1: well anterior to level of anus (>five annuli anterior to anus), 2: at level of anus (from five annuli anterior to five annuli posterior to anus), 3: well posterior to level of anus (>five annuli posterior to anus).

In order to facilitate the identification of *Rotylenchus* spp., based on a cluster analyses a web-based key was developed. The domain for this web-based key was registered from the website: www.awardspace.com. The interface of this web-based key was written using Notepad++ v7.5.6 and the algorithm was based on Bray–Curtis similarity measure. The web-based key can be freely accessed at http://nematodeidentification.mypressonline.com/category/identification-tool/.

## Results

SYSTEMATICS

*Rotylenchus rhomboides* n. sp.

**Figure 1:**
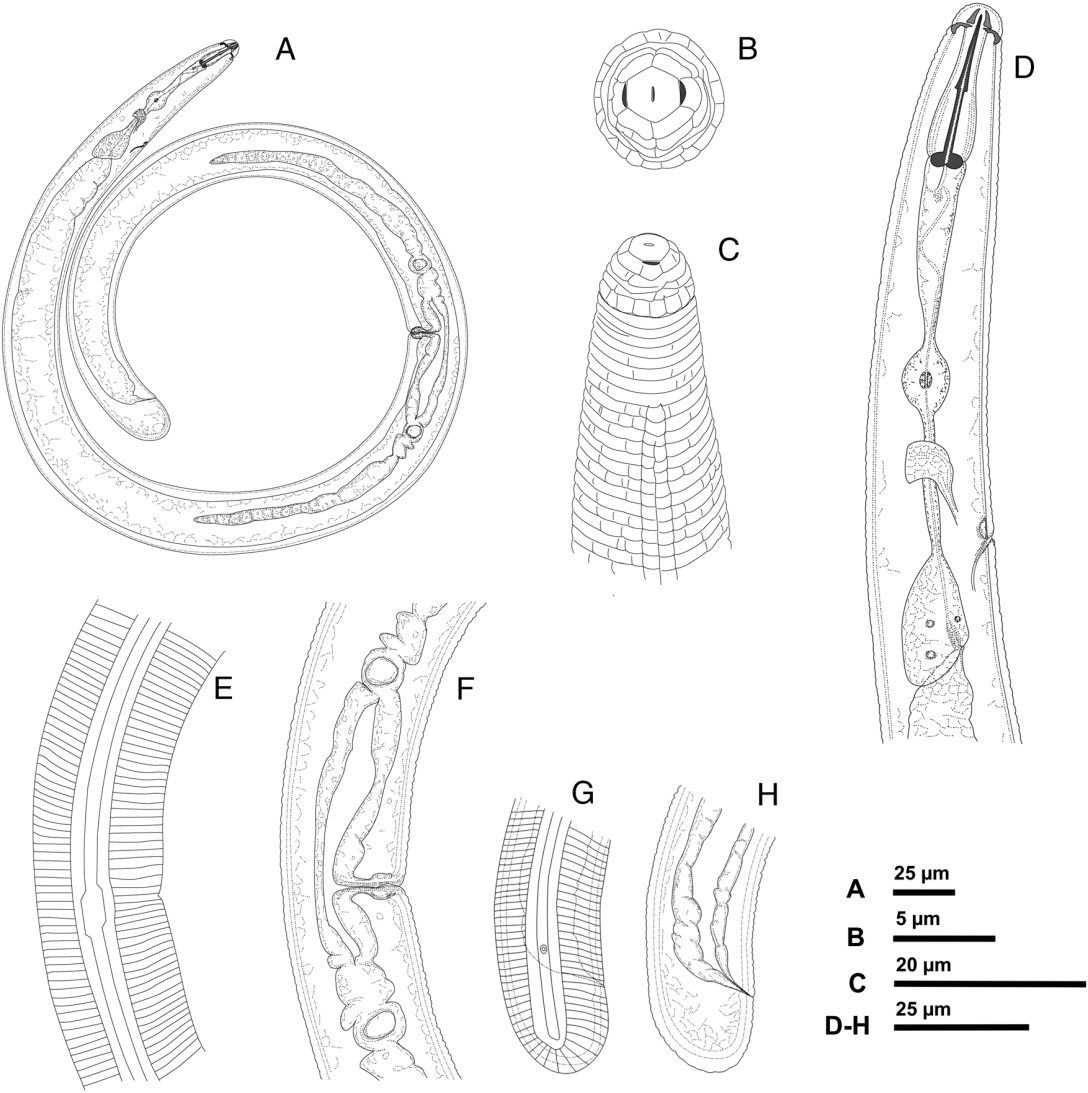
*Rotylenchus rhomboids* n. sp., (drawings ♀). (A) entire body; (B) en face view; (C) head region; (D) esophageal region; (E) lateral field at vulva region; (F) vulva region; (G) tail region; (H) lateral field at tail region. (B, C based on SEM illustrations) (A, E, F, G, H: Holotype) (Scale bar: A: 25 µm; B: 5 µm; C: 20 µm; D–H: 25 µm).

**Figure 2:**
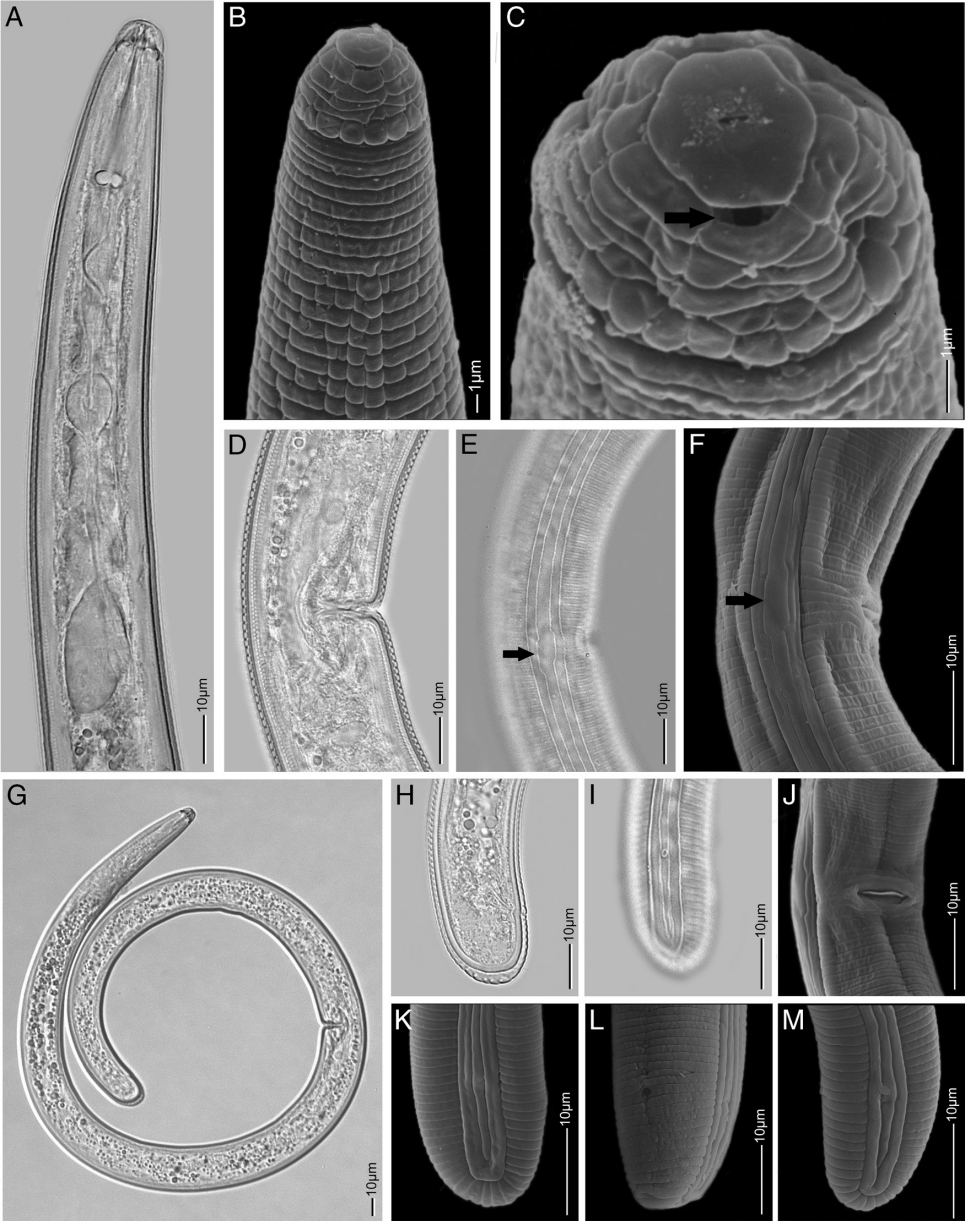
*Rotylenchus rhomboides* n. sp. (LM ♀). (A) esophageal region; (D) vulva region; (E) lateral field at vulva region with a rhomboid widening (see arrow); (G) entire body; (H) tail region; (I) lateral field with phasmid at tail region (SEM ♀); (B) anterior body region, lateral view; (C) en face view (arrow indicates amphid aperture); (F) vulva region, lateral view; (J) vulva region, ventral view; (K) tail region of female, lateral view; (L) tail region of female, oblique ventral view; (M) tail region of juveniles (D, E, G, H, I: Holotype; others paratypes) (Scale bar: B, C: 1 µm; A, D–M: 25 µm).

**Figure 3:**
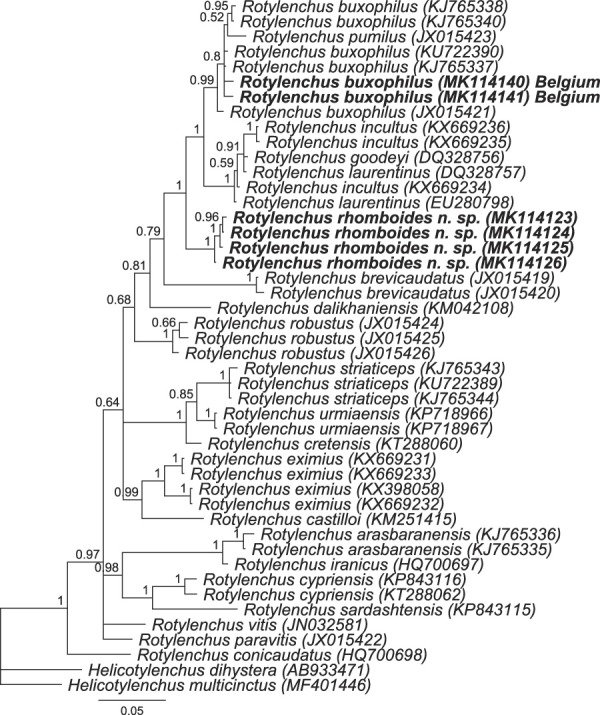
BI phylogenetic tree generated from the D2-D3 of 28S rDNA region data set with the HKY + G model (94 parameters). Bayesian posterior probabilities are given next to each node. Sequences of *Rotylenchus rhomboides* n. sp. and *R. buxophilus* (Belgium) are in bold.

**Figure 4:**
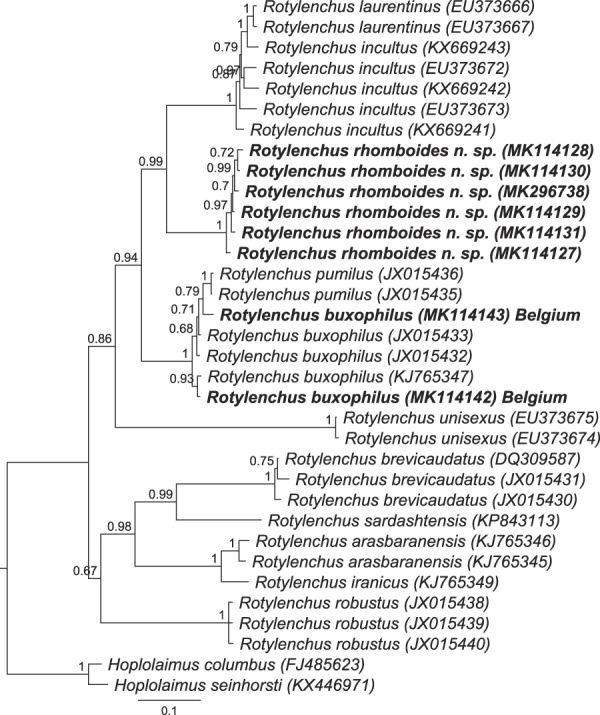
BI phylogenetic tree generated from ITS sequences with HKY + G model (70 parameters). Bayesian posterior probabilities are given next to each node. Sequences of *Rotylenchus rhomboides* n. sp. and *R. buxophilus* (Belgium) are in bold.

**Figure 5:**
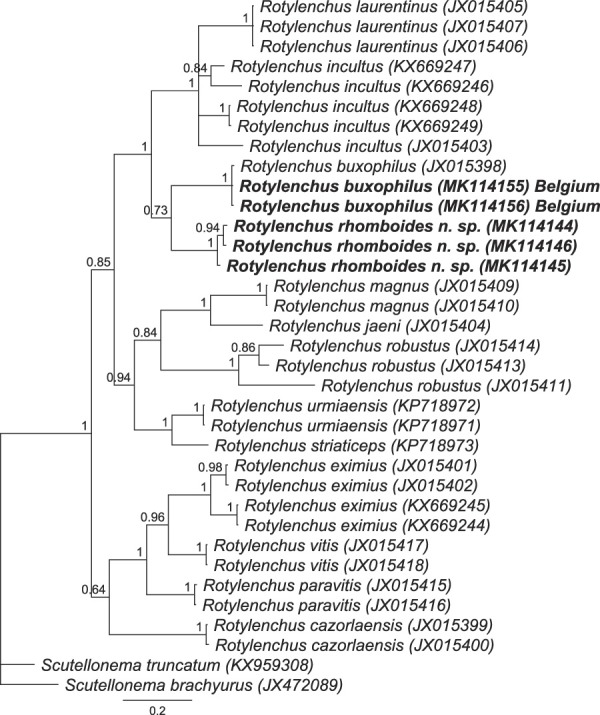
BI phylogenetic tree generated from *COI* sequences with the GTR +G + I model (77 parameters). Bayesian posterior probabilities are given next to each node. Sequences of *Rotylenchus rhomboides* n. sp. and *R. buxophilus* (Belgium) are in bold.

**Figure 6:**
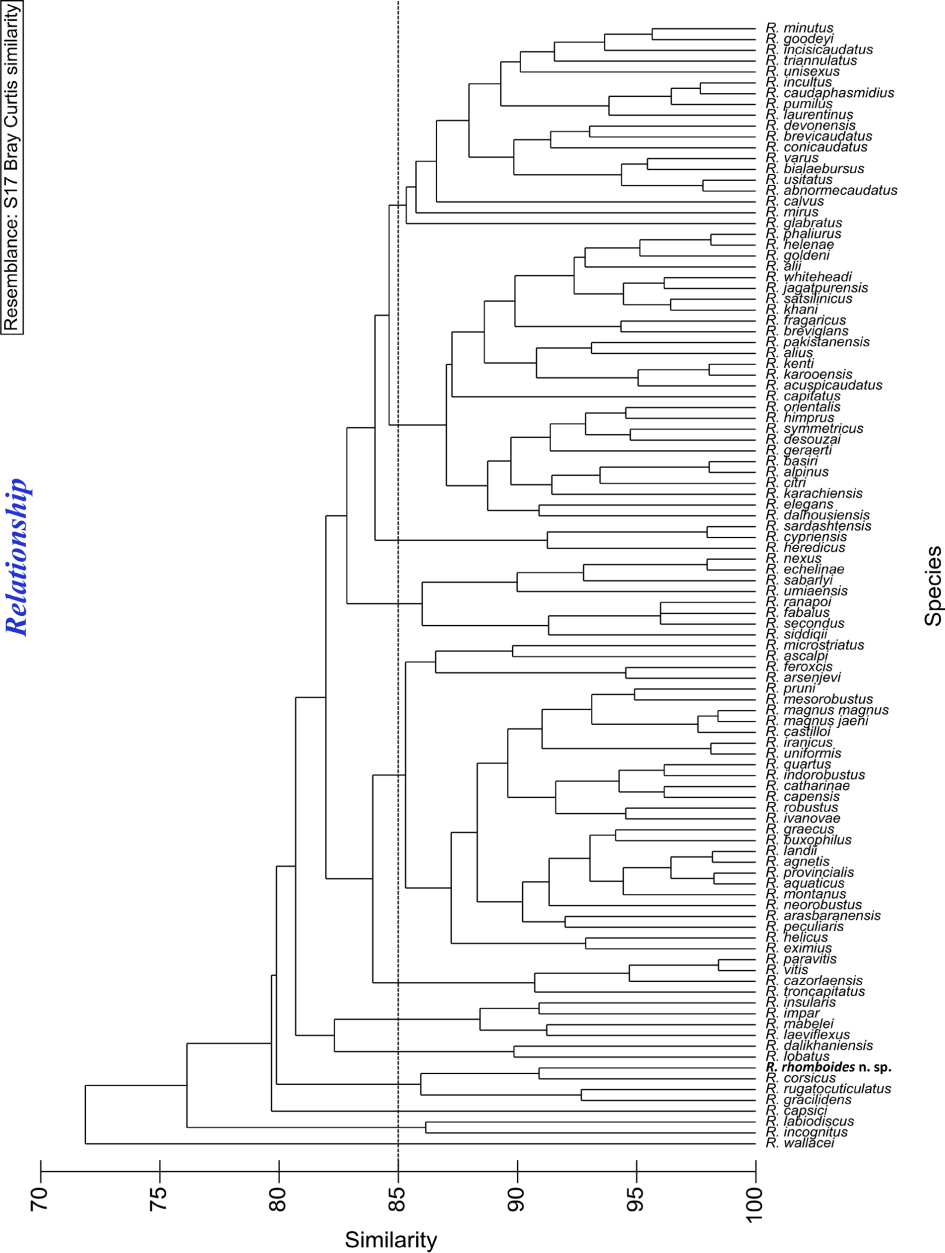
The cluster analysis of 104 species (included *Rotylenchus* n. sp.) based on Bray–Curtis similarity measure of 11 ranked features. *Rotylenchus rhomboides n. sp.* is in bold. The dotted line indicates 85% similarity.

Figures 1–6, Table 1–3.

**Figure 7:**
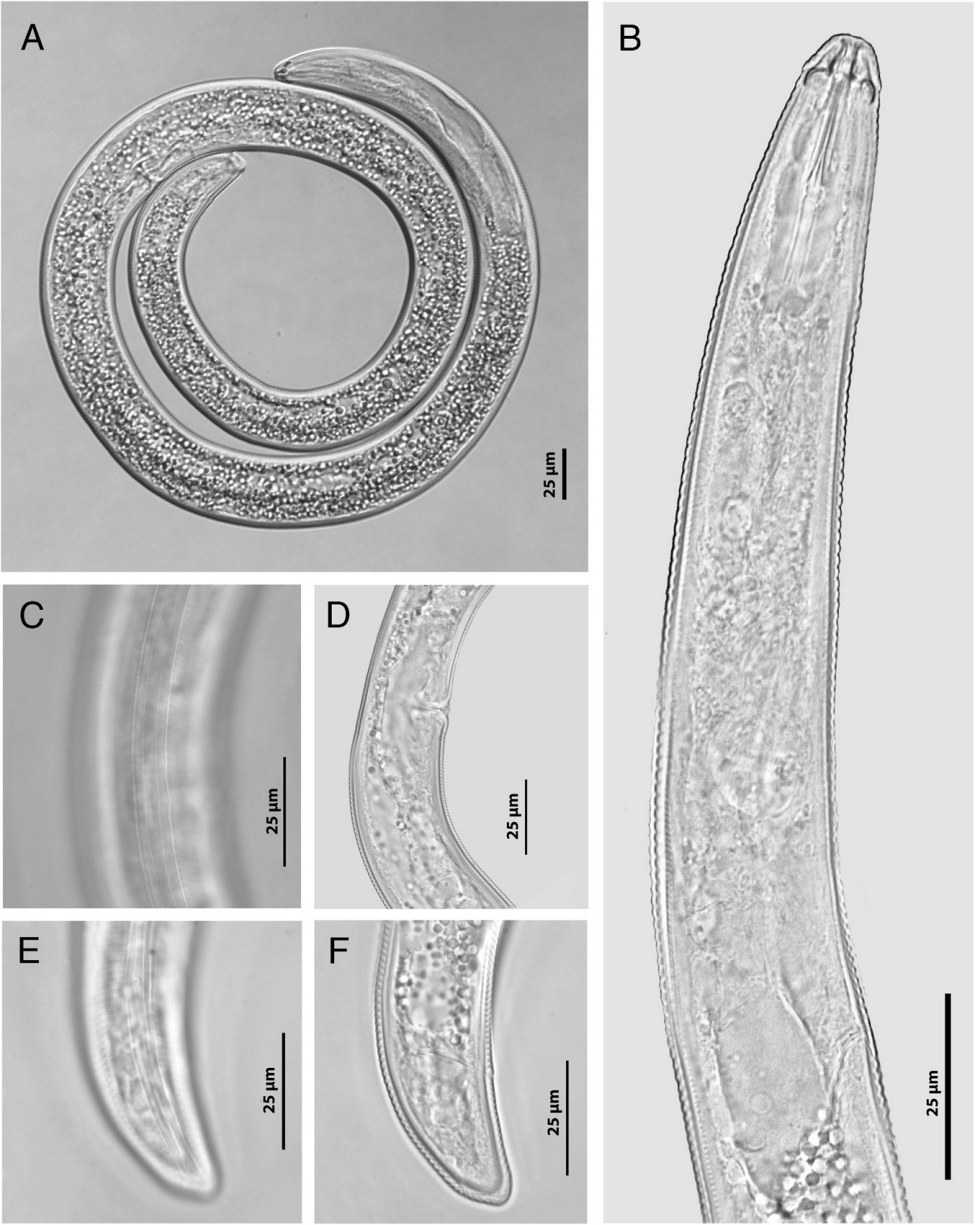
Belgian population of *Rotylenchus buxophilus* (LM ♀). (A) entire body; (B) esophageal region; (C) lateral field at vulva region; (D) vulva region; (E) lateral field with phasmid at tail region; (F) tail region (Scale bar: A–F: 25 µm).

**Table 1:** Morphometric data of *Rotylenchus rhomboides n*. sp. All measurements are in μm (except for ratio) and in the form: mean ± sd (range).

Character	Holotype female	Paratype females
*n*	1	12
Body length (L)	827	861 ± 101 (634–1049)
a = L/MBD	28.5	31.8 ± 2.8 (27.8–35.1)
b’ = L/Anterior end to the end of esophageal gland	7.6	6.9 ± 0.5 (5.6–7.6)
c = L/Tail length	55	63.3 ± 10.7 (50.3–85.5)
c’ = Tail length/ABD	68	0.7 ± 0.1 (0.5–0.8)
V = Anterior end to vulva/L × 100	59	56 ± 2.6 (53–61)
Lip height	6	6 ± 0.7 (5–7)
Lip width	11	10 ± 0.8 (8–11)
Stylet length	35	33 ± 1.5 (31–37)
Conus length	17	17 ± 0.8 (16–18)
Shaft length	14	13 ± 0.9 (12–14)
Knob height	3	3 ± 0.4 (3–4)
Dorsal gland opening from stylet base	4	3 ± 0.9 (2–5)
Anterior end to secretory-excretory pore	100	107 ± 10.6 (77–123)
Anterior end to nerve ring	78	86 ± 9.9 (60–96)
Anterior end to esophageal gland end	108	125 ± 10.5 (108–146)
Esophagus overlapping	12	14 ± 2.3 (9–19)
Max body diameter (MBD)	29	27 ± 2.3 (23–31)
Anal body diameter (ABD)	22	20 ± 1.8 (16–23)
Tail length	15	14 ± 2.0 (9–16)
Tail annuli number	11	10 ± 1.0 (8–11)

**Table 2:** Comparison of the matrix code of *Rotylenchus rhomboides* n. sp. with other closely related species.

Species	A	B	C	D	E	F	G	H	I	J	K
*Rotylenchus rhomboides* n. sp.	3–4	2	1	2	2	2	2	1–2	2	2	2
*R. buxophilus* from Belgium	4	1	1	4	2	2	3	3	2	2	1
*R. buxophilus*	4	1	1	4	2	2	3	3	2	2	1
*R. corsicus*	4	1	1	2	2	2	2	3	2	2	1
*R. goodeyi*	3	1	1	4	2	2	4	2	2	1	2
*R. gracilidens*	3	1	1	2	2	2	3	1	2	1	1
*R. incultus*	4	1	1	4	1	2	3	1	2	1	1
*R. laurentinus*	4	1	1	3	2	2	3	1	2	1	2
*R. pumilus*	4	1	1	4	1	2	2	1	2	1	2
*R. rugatocuticulatus*	3	2	1	2	3	2	4	1	2	1	1

**Table 3:** Rotylenchus species and populations used and sequenced in the present study.

	Accession number				
Species	28S	ITS	*COI*	Host	Locality
*H. dihystera*	AB933471			Sugarcane	Japan
*H. multicinctus*	MF401446			Banana	Greece
*H. columbus*		FJ485623		Unknown	USA
*H. seinhorsti*		KX446971		Unknown	China
*R. arasbaranensis*	KJ765335	KJ765345		Grasses	Iran
	KJ765336	KJ765346		Grasses	Iran
*R. brevicaudatus*	JX015419			Grasses	Australia
	JX015420			Grasses	Australia
		JX015431		Grasses	Australia
*R. buxophilus*	JX015421			–	USA
	KJ765337			Euonymus sp.	Iran
	KJ765338			Euonymus sp.	Iran
	KJ765340			Euonymus sp.	Iran
	KU722390			–	Iran
		JX015432		Unknown	USA
		JX015433		Unknown	USA
		KJ765347		Euonymus sp.	Iran
***R. buxophilus***	**MK114140**	**MK114142**	**MK114155**	***Dioscorea tokoro***	**Belgium**
	**MK114141**	**MK114143**	**MK114156**	***Dioscorea tokoro***	**Belgium**
*R. castilloi*	KM251415			*Fagus orientalis* Lipsky (oriental beech tree)	Iran
*R. cazorlaensis*			JX015399	*Quercus faginea* (Portuguese oak)	Spain
			JX015400	*Quercus faginea* (Portuguese oak)	Spain
*R. conicaudatus*	HQ700698			Grasses	Iran
*R. cretensis*	KT288060			Olive	Greece
*R. cypriensis*	KP843116			*Prunus* sp.	Iran
	KT288062			Olive	Greece
*R. dalikhaniensis*	KM042108			*Ruscus hyrcanus* Woronow	Iran
*R. eximius*	KX398058			*Tamarix smyrnensis*	Greece
	KX669231		KX669244	Olive	Tunisia
	KX669232			Olive	Tunisia
	KX669233		KX669245	Olive	Tunisia
			JX015401	*Pistacia lentiscus* (lentisc)	Italy
			JX015402	*Pistacia lentiscus* (lentisc)	Italy
*R. goodeyi*	DQ328756			*Olea europaea* subsp. silvestris (wild olive)	Spain
*R. incultus*	KX669234			Olive	Tunisia
	KX669235			Olive	Tunisia
	KX669236			Olive	Tunisia
			JX015403	*Vitis vinifera* (grapevine)	Spain
			KX669246	Olive	Tunisia
			KX669247	Olive	Tunisia
			KX669248	Olive	Tunisia
			KX669249	Olive	Tunisia
*R. iranicus*	HQ700697			Unidentified forest tree	Iran
*R. jaeni*			JX015404	*Quercus suber* (cork tree)	Spain
*R. laurentinus*	DQ328757		JX015405	*Pistacia lentiscus* (lentisc)	Italy
			JX015406	*Pistacia lentiscus* (lentisc)	Italy
			JX015407	*Pistacia lentiscus* (lentisc)	Italy
	EU280798			*Pistacia lentiscus* (lentisc)	Spain
*R. magnus*			JX015409	*Ilex aquifolium* (holly)	Spain
			JX015410	*Ilex aquifolium* (holly)	Spain
*R. paravitis*	JX015422		JX015415	*Helianthus annuus* (sunflower)	Spain
			JX015416	*Helianthus annuus* (sunflower)	Spain
*R. pumilus*	JX015423	JX015435		*Urtica* sp.	USA
		JX015436		*Urtica* sp.	USA
***R. rhomboides*** **n. sp.**	**MK114123**	**MK114127**	**MK114144**	***Musa basjoo***	**Belgium**
	**MK114124**	**MK114128**	**MK114145**	***Musa basjoo***	**Belgium**
	**MK114125**	**MK114129**	**MK114146**	***Musa basjoo***	**Belgium**
	**MK114126**	**MK114130**		***Musa basjoo***	**Belgium**
		**MK114131**		***Musa basjoo***	**Belgium**
		**MK296738**		***Musa basjoo***	**Belgium**
*R. robustus*	JX015424		JX015413	*Pinus pinea* (stone pine)	Spain
	JX015425	JX015438		Grasses	USA
	JX015426	JX015439	JX015411	Grasses	USA
		JX015440		Grasses	USA
			JX015414	*Pinus pinea* (stone pine)	Spain
*R. sardashtensis*	KP843115	KP843113		Unidentified grasses	Iran
*R. striaticeps*	KJ765343			Unidentified forest tree	Iran
	KJ765344			Unidentified forest tree	Iran
	KU722389			Unknown	Iran
			KP718973	Unknown	Iran
*R. unisexus*		EU373674		*Citrus aurantium* (citrus)	Spain
		EU373675		*Citrus aurantium* (citrus)	Spain
*R. urmiaensis*	KP718966		KP718971	*Corylus* spp.	Iran
	KP718967		KP718972	*Corylus* spp.	Iran
*R. vitis*	JN032581		JX015417	*Vitis vinifera* (grapevine)	Spain
			JX015418	*Vitis vinifera* (grapevine)	Spain
*S. brachyurus*			JX472089	*Sansevieria laurentii*	USA
*S. truncatum*			KX959308	Unknown	USA

Note: Newly added sequences are indicated by bold font.

### Description

#### Females

Body relatively small, habitus spiral or C-shaped (Figs 1A, 2G). Cuticle clearly annulated with irregular longitudinal striations in anterior region; annuli 1.6 to 2 μm wide at mid-body. Lateral fields with four lines at mid-body, beginning anteriorly at 7 to 8th annulus as two lines forming one band, third line appearing at level 10 to 11th annulus; mid-ridge at vulva level with a characteristic rhomboid widening. Regular areolation of lateral fields observed only in esophageal region (Figs 1C, E, 2B, E, F). Labial region rounded, offset from rest of body, bearing 4 to 5 annuli, divided longitudinally; labial disc rounded to hexagonal, marked from rest of labial region, but not elevated (Figs 1B, C, 2B, C). Stylet robust; basal knobs rounded, 3 to 4 μm high. Dorsal esophageal gland opening 2 to 5 μm posterior to stylet base. Procorpus cylindrical, with slight depression just anterior to median bulb; median bulb well developed, rounded, or broadly oval; isthmus slender, encircled by nerve ring; esophageal glands sacciform, overlapping intestine dorsally. Secretory-excretory pore usually located just posterior to the hemizonid. Hemizonid distinct *ca* 1.5 to 2 body annuli long, located around esophago-intestinal junction level (Figs 1D, 2A). Reproductive system didelphic-amphidelphic, genital branches equally developed; vulva slightly posterior to mid-body, without distinct epiptygma; outstretched ovaries with a single row of oocytes; spermatheca mostly oval, without sperm (Figs 1A, F, 2D, F, G). Tail short, varying in shape from hemispherical to rounded, with 8 to 11 annuli ventrally; terminus striated. Phasmid opening relatively large 1.6 (1.2–2) μm, pore-like, located 3 to 5 annuli anterior to level of anus (Figs 1G, H, 2H, I, K, L).

#### Male

Not found.

### Molecular characterization

#### 28S rDNA

Four sequences of 5′-end region of 28S rDNA were obtained, 916 to 1057 bp long, with an intraspecific variation of 1 to 4 positions (0.1–0.5%). The muscle alignment comprised 46 sequences, 1,080 positions. The sequences of D2-D3 of 28S rDNA *Rotylenchus rhomboides* n. sp. differ by 24 to 74 positions (90–96% similar) compared to the sequences of all other *Rotylenchus* species in this study. These sequences were most similar to the sequences of *R. buxophilus* with 24 to 28 different positions (95–96% similar). The Bayesian interference phylogenetic tree based on the D2-D3 of 28S rDNA sequences showed that the sequences of *Rotylenchus rhomboides* n. sp. have a maximally supported sister relation with *R. buxophilus*, *R. pumilus* (Perry et al., 1959; Sher, 1961), *R. incultus* (Sher, 1965), *R. goodeyi*, and *R. laurentinus* (Scognamiglio and Talamé, 1972) (Fig. 3).

#### ITS rDNA

Six ITS rDNA sequences were obtained, 1,026 to 1,339 bp long, with differences on 0 to 4 nucleotides (99–100% similar). The muscle alignment included 34 nucleotide sequences and 1,444 positions. The sequences of *Rotylenchus rhomboides* n. sp. differed by 158 to 357 positions (60–83% similar) compared to all other *Rotylenchus* species in this study. The sequences of *Rotylenchus rhomboides* n. sp. are most similar to the sequence of *R. buxophilus* with 138 to 210 different positions (80–83% similar). The ITS tree topology showed small changes compared to D2-D3 tree topology. The sequences of *Rotylenchus rhomboides* n. sp. have a sister relationship to *R. incultus* and *R. laurentinus* (0.97 PP) (Fig. 4).

#### *COI* mtDNA

Three *COI* mtDNA sequences were obtained (442 bp long). The sequences of *Rotylenchus rhomboides* n. sp. varied 5 to 8 positions (2–3% different) compared to each other. The muscle alignment consisted of 35 nucleotide sequences, 445 positions long. The sequences of *Rotylenchus rhomboides* n. sp. differed by 47 to 101 positions (72–86% similar) compared to other *Rotylenchus* species in this study. The sequences of *Rotylenchus rhomboides* n. sp. are most similar to the sequence of *R. buxophilus* with 47 to 48 different positions (85–86% similar). The phylogenetic tree based on the *COI* mtDNA showed few changes compared to the D2-D3 of 28S and ITS rDNA trees. In that, the sequences of *Rotylenchus rhomboides* n. sp. also have a maximal sister relation to *R. laurentinus, R. incultus*, and *R. buxophilus*, but there were few changes in the position of each species (Fig. 5).

### Diagnosis and relationships

*Rotylenchus rhomboides* n. sp. is characterized by a combination of the following traits: a rounded labial region with four annuli; an annulated body cuticle with irregular longitudinal striations in the anterior region; lateral field with four lines forming three ridges at mid-body, areolated only at esophageal region; a rhomboid widening at the mid-ridge at level of the vulva; a robust stylet of average length (31–37 μm); esophageal glands shortly overlapping the intestine dorsally; female reproductive system with oval spermatheca without sperm and a vulva located slightly posterior to mid-body; hemispherical or rounded tail tip with relatively large phasmids (1.2–2 µm) located 3 to 5 annuli anterior to the level of the anus; male not found. According to Castillo and Vovlas (2005), the matrix code for this new species is: A3–4, B2, C1, D2, E2, F2, G2, H1-2, I2, J2, and K2. *Rotylenchus rhomboides* n. sp. differs from all *Rotylenchus* species by the presence of the rhomboid widening of the mid-ridge of the lateral field at vulva level.

*Rotylenchus rhomboides* n. sp. is also different from all other species according to the dichotomous key of Castillo and Vovlas (2005) as well as the comparison with other species from the studies of Vovlas et al. (2008), Atighi et al. (2011), Cantalapiedra-Navarrete et al. (2012, 2013), Talezari et al. (2015), and Golhasan et al. (2016). By using the hierarchical cluster analysis of all the characters that were used in the tabular key of Castillo and Vovlas (2005), all 103 valid species and *Rotylenchus rhomboides* n. sp. were separated into groups that have the highest similarities. This cluster analyses can also be done by using web-based key at: http://nematodeidentification.mypressonline.com/category/identification-tool/. The new species was grouped together with the most similar species, namely *R. corsicus*, *R. gracilidens* (Sauer, 1958), and *R. rugatocuticulatus* (Sher, 1965) (Fig. 6). They shared more than 85% similarity to each other and less than 80% similarity to all other species. The shared characteristics of this group are the labial region having 4 to 5 annuli, body longitudinal striations being restricted to the esophageal region, DGO at 2 to 6.9 μm from the base of the stylet and ratio V between 50 and 70%.

However, *Rotylenchus rhomboides* n. sp. can be distinguished from *R. corsicus* by 3 out of the 11 main characters used in the tabular key of Castillo and Vovlas (2005), including B: labial region shape (rounded vs hemispherical); H: tail shape (rounded to hemispherical without mucron vs conoid to rounded with mucron in some specimens); K: phasmid position (located 3–5 annuli anterior to anus level vs 5–16 annuli anterior to anus level) (Table 2). Morphometrically, *Rotylenchus rhomboides* n. sp. differs from *R. corsicus* by having a smaller maximum body diameter (23–31 μm vs 48–57 μm) and smaller c’ value (0.5–0.8 vs 1.0–1.3).

*Rotylenchus rhomboides* n. sp. differs from *R. gracilidens* by 4 out of 11 compared characters in tabular key, including B: Labial region shape (rounded labial region vs hemispherical labial region); G: Dorsal esophageal gland overlapping (between 6 and 20.9 μm vs between 21 and 30.9 μm); J: Presence of males (absent vs present); K: phasmid position (phasmids 3–5 annuli anterior to the anus level vs 15–20 annuli anterior to the anus level).

*Rotylenchus rhomboides* n. sp. differs from *R. rugatocuticulatus* by 4 out of 11 compared characters in tabular key, including E: stylet length (between 30 and 35.9 µm vs between 36 and 40.9 µm); G: dorsal esophageal gland overlapping (between 6 and 20.9 μm vs between 31 and 40.9 μm); J: Presence of males (absent vs present); K: phasmid position (phasmids 3–5 annuli anterior to the anus level vs 8–10 annuli anterior to the anus level). Moreover, *Rotylenchus rhomboides* n. sp. has different vulva structure (without epiptygma vs double epiptygma), a smaller body length (0.6–1 mm vs 1–1.3 mm).

*Rotylenchus rhomboides* n. sp. is phylogenetically close to *R. buxophilus*, *R. goodeyi, R. laurentinus, R. pumilus*, and *R. incultus*. *Rotylenchus rhomboides* n. sp. can also be differentiated from the phylogenetically closely related species by the morphological features. *Rotylenchus rhomboides* n. sp. differs from *R. buxophilus* by 5 out of 11 compared characters in the tabular key including B: Labial region shape (rounded labial region vs hemispherical labial region), D: Body longitudinal striations (longitudinally striated in esophageal region vs without body striation), G: Dorsal esophageal gland overlapping (between 6 and 20.9 μm vs between 21 and 30.9 μm), H: tail shape (rounded to hemispherical vs dorsally convex-conoid), K: phasmid position (phasmids 3–5 annuli anterior to the anus level vs 6–13 annuli anterior to anus level). *Rotylenchus rhomboides* n. sp. also has a smaller body length (861 μm vs 1090 μm), and larger c value (63.3 vs 43).

*Rotylenchus rhomboides* n. sp. differs from *R. goodeyi* by 4 out of 11 compared characters in tabular key of Castillo and Vovlas (2005), including B: Labial region shape (rounded labial region vs hemispherical labial region); D: Body longitudinal striations (longitudinally striated in esophageal region vs without body striation); G: Dorsal esophageal gland overlapping (between 6 and 20.9 μm vs between 31 and 40.9 μm); J: Presence of males (absent vs present). *Rotylenchus rhomboides* n. sp. can also be differentiated from *R. goodeyi* by having different number of lip annuli (4–5 annuli vs 3–4 annuli), smaller body length (0.86 mm vs 0.97 mm), different phasmid position (phasmids 3–5 annuli anterior to the anus level vs 1–11 annuli anterior to anus level).

*Rotylenchus rhomboides* n. sp. differs from *R. laurentinus* by 4 out of 11 compared characters in tabular key of Castillo and Vovlas (2005), including B: Labial region shape (rounded labial region vs hemispherical labial region); D: Body longitudinal striations (longitudinally striated in esophageal region vs longitudinally striated over whole body); G: Dorsal esophageal gland overlapping (between 6 and 20.9 μm vs between 21 and 30.9 μm); J: Presence of males (absent vs present). *Rotylenchus rhomboides* n. sp. also differs from *R. laurentinus* by having a vulva without distinct epiptygma vs vulva with posterior epiptygma present overlap less conspicuous anterior one.

*Rotylenchus rhomboides* n. sp. differs from *R. pumilus* by 4 out of the 11 main compared characters in tabular key including B: labial region shape (rounded vs hemispherical); D: Body longitudinal striations (longitudinally striated in esophageal region vs without body striation), E: stylet length (31–37 μm vs 23–28 μm); J: Presence of males (absent vs present).

*Rotylenchus rhomboides* n. sp. differs from *R. incultus* by 6 out of the 11 main compared characters in the tabular key including B: labial region shape (rounded vs hemispherical); D: Body longitudinal striations (longitudinally striated in esophageal region vs without body striation); E: stylet length (31–37 μm vs 24–28 μm); J: Presence of males (absent vs present); K: phasmid position (located 3–5 annuli anterior to anus level vs 13–14 annuli anterior to anus level).

### Etymology

Name of the new species regarded from rhomboideus (modern Latin), which refers to typical rhomboid enlargement in the lateral field at vulva level.

### Type host and locality

*Rotylenchus rhomboides* n. sp. was recovered from soil and root samples from the rhizosphere of banana (*Musa basjoo*) in the botanical garden of Ghent University, Belgium (GPS coordinates N: 51°2′6.8″, E: 3°43′22.7″).

### Type material

Holotype female and four paratype females, all in one slide, are deposited at the Ghent University Museum, Zoology Collections, collection number UGMD 104367. Four paratype females in one slide were deposited at National Plant Protection Organization, Wageningen Nematode Collection (WaNeCo). Additional paratypes (five females and three juveniles in two slides) are available in the UGent Nematode Collection (slide UGnem-189–190) of the Nematology Research Unit, Department of Biology, Ghent University, Ghent, Belgium. ZooBank ID: urn:lsid:zoobank.org:act:EF3FEED6-E804-4EFE-BCC2-12CBF072ADA3.

***Rotylenchus buxophilus*** (Golden, 1956)

Figures 3–5, 7, Tables 4, 5.

**Table 4:** Morphometric data of *Rotylenchus buxophilus* from Belgium. All measurements are in μm (except for ratio) and in the form: mean ± sd (range).

Character	Females
*n*	15
Body length (L)	972 ± 87 (818–1065)
a = L/MBD	30.1 ± 1.9 (27.2–32.5)
b′ = L/Anterior end to the end of esophageal gland	7.0 ± 0.8 (5.3–8.2)
c = L/Tail length	40.1 ± 4.1 (34.6–49.8)
c′ = Tail length/ABD	1.2 ± 0.1 (1.0–1.4)
V = Anterior end to vulva/L × 100	53 ± 3 (48–58)
Lip height	7 ± 0.3 (6–8)
Lip width	11 ± 0.6 (10–12)
Stylet length	36 ± 1.6 (32–38)
Conus length	18 ± 1.1 (16–20)
Shaft length	14 ± 0.7 (13–14)
Knob height	4 ± 0.6 (3–5)
Dorsal gland opening from stylet base	4 ± 1.2 (3–7)
Anterior end to secretory-excretory pore	123 ± 10 (105–139)
Anterior end to nerve ring	99 ± 7 (87–109)
Anterior end to esophageal gland end	140 ± 11 (124–157)
Esophagus overlapping	22 ± 5 (18–28)
Max body diameter (MBD)	32 ± 2 (29–37)
Anal body diameter (ABD)	21 ± 1.6 (19–24)
Tail length	24 ± 3.1 (18–28)
Tail annuli number	14 ± 1.3 (12–16)

**Table 5:** The measurements of known and new populations of *Rotylenchus buxophilus*.

Female	*R. buxophilus* (Belgian population)	*R. buxophilus* Golden (1956)	*R. buxophilus* Sher (1965)	*R. buxophilus = R. sheri* Jairajpuri (1963)	*R. buxophilus* Geraert and Barooti (1996)	*R*. *buxophilus* Wouts and Sturhan (1999)
Host, location	Yam (*Dioscorea tokoro*), Belgium	English boxwood (*Buxus sempervirens* var. *suffruticosa* L.), USA	camellia (*Camellia japonica* L.), France	*Cedrus libani* var*. deodara*, India	*Magnolia* sp. L., Iran	Cabbage tree (*Cordyline* sp. Comm, ex R.Br.), New Zealand
*n*	15	20	20	10	4	6
L	972 (818–1065)	1,090	920–1,310	900–1,200	850–1,070	1,020–1,250
V	53 (48–58)	55	52–58	52–56	54–58	55–58
STL	36 (32–38)	33.5	34–38	35–39	34	38–40
a	30.1 (27.2–32.5)	31	28–38	27–35	25–32	32–43
b	7.0 (5.3–8.2)	7	6.4–8.9	5.5–7.0	6–8	7.3–8.7
c	40.1 (34.6–49.8)	43	36–48	38–47	33–45	41–58
c′	1.2 (1.0–1.4)	–	–	–	1.1–1.4	0.8–1.2

### Morphological characterizations of *Rotylenchus buxophilus* in Belgium

#### Females

Body relatively large, habitus mostly spiral (Fig. 7A). Cuticle clearly annulated. Lateral field with four lines at mid-body, areolated at esophageal region (Fig. 7B, C). Labial region hemispherical, bearing five annuli. Stylet robust; basal knobs rounded. Median bulb rounded to oval; isthmus slender, encircled by nerve ring; esophageal glands sacciform, overlapping intestine dorsally 18–28 μm. Secretory-excretory pore anterior to esophago-intestinal junction level; hemizonid distinct *ca* 1.5 to 2 body annuli long, just anterior to secretory-excretory pore (Fig. 7B). Reproductive system didelphic-amphidelphic, branches equally developed; outstretched ovaries with a single row of oocytes; vulva at mid-body level, with double distinct epiptygma; spermatheca inconspicuous, without sperms (Fig. 7A, D). Tail dorsally convex-conoid shape; pore-like phasmids located 9 to 16 annuli anterior to anus level (Fig. 7E, F).

#### Male

Not found.

### Molecular characterization

All the molecular analyses of the Belgian population of *Rotylenchus buxophilus* were executed together with the sequences of *Rotylenchus rhomboides* n. sp. as above.

#### 28S rDNA

Two sequences of 5′-end region of 28S rDNA were obtained (844 and 1,047 bp long). These sequences differ from other sequences of *R. buxophilus* (accession numbers: KJ765338, KJ765340, KJ765337, and KU722390) by 1 to 4 nucleotides (99–100% similar) and they differ from other *Rotylenchus* species by 9 to 71 nucleotides (85–97% similar). In the resulting phylogenetic tree, *R. buxophilus* from Belgium is in a well-supported clade (0.99 PP) together with all other sequences of *R. buxophilus* and *R. pumilus* (Fig. 3).

#### ITS rDNA

Two sequences of ITS rDNA sequences were obtained, 1,175 and 1,249 bp long. The ITS sequences of the Belgian population of *R. buxophilus* differ from other sequences of *R. buxophilus* (accession numbers: KJ765347, JX015435, JX015432, and JX015433) by 6 to 7 nucleotides (97–98% similar). The interspecific differences to other *Rotylenchus* sequences are 158 to 339 nucleotides (66–81% similar). *Rotylenchus buxophilus* from Belgium has a maximal relationship to other sequences of *R. buxophilus* and *R. pumilus* (Fig. 4).

#### *COI* mtDNA

Two sequences of *COI* mtDNA sequences were obtained, 444 and 445 bp long. The *COI* mtDNA sequences of the Belgian population of *R. buxophilus* are identical to the sequence of *R. buxophilus* on GenBank (accession number: JX015398) and these sequences differ from other *Rotylenchus* species in this study by 50 to 103 nucleotides (72–88% similar). *Rotylenchus buxophilus* from Belgium was placed in a maximal supported clade together with other sequences of *R. buxophilus* (Fig. 5).

### Remarks

The morphological characteristics of the Belgian population of *R. buxophilus* resemble the original description of *R. buxophilus*. The few minor variations were observed, including the number of lip annuli (5 annuli vs 4–5 annuli), V value (48–58 vs 55), stylet length (32–38 μm vs 33.5 μm), a value (27.2–32.5 vs 31), and c value (34.6–49.8 vs 43). However, these differences are insignificant and the measurements of other population also show some variations (Table 5).

According to Castillo and Vovlas (2005), the matrix code for this population is: A4, B1, C1, D4, E2, F2, G3, H3, I2, J2, and K1. This matrix code is identical to that of the type population of *R. buxophilus*.

The BI phylogenetic trees on Figures 3–5 showed a very close relationship of the sequences from our population compared to the sequences of *R. buxophilus* and *R. pumilus* from GenBank. However, our population can be differentiated from *R. pumilus* by 3 out of 11 compared characters in tabular key of Castillo and Vovlas (2005), including E: stylet length (32–38 μm vs 23–28 μm); H: tail shape (conoid vs hemispherical); J: presence of males (absent *vs* present); K: phasmid position (located 9–16 annuli anterior to anus level *vs* located immediately posterior to latitude of anal opening). Furthermore, the females of our population have a larger body length (818–1065 µm vs 600–700 µm), smaller V value (48–58 vs 58–64), and larger a value (27.2–32.5 vs 20.0–23.7).

### Host and locality

The Belgian population of *Rotylenchus buxophilus* was recovered from soil and root samples from the rhizosphere of Yam (*Dioscorea tokoro*) in the botanical garden of Ghent University, Belgium (GPS coordinates: N: 51°2′6.9″, E: 3°43′22.6″).

### Voucher specimens

All the permanent slides (19 females in 3 slides) are available in the UGent Nematode Collection (slide number: UGnem-191–193) of the Nematology Research Unit, Department of Biology, Ghent University, Ghent, Belgium.

## Discussion

The identification keys for *Rotylenchus* spp. used to be dichotomous (Sher, 1965; Geraert and Barooti, 1996). However, according to Geraert and Barooti (1996), the overlapping of various character states makes species differentiation difficult, something that represents an especially great challenge with genera with a large number of species (104 valid species at present for the genus *Rotylenchus*). Based on the principle of polytomous keys, Castillo and Vovlas (2005) developed a tabular or matrix key for the genus *Rotylenchus*, which facilitates the identification process and includes both major and supplementary characters for the identification of *Rotylenchus* species, currently in use. In this study, the utilization of hierarchical cluster analysis using the software Primer 6 with the data set based on the tabular key principle was successful in separating the 104 species including *Rotylenchus rhomboides* n. sp. into small groups that shared the highest number of similarities. This application can speed up the identification process, avoid mistakes made in manual comparisons (especially for a large data set), and avoid biases in the selection of closely related species to compare with. Furthermore, our web-based key facilitates the cluster analysis and is freely available at: http://nematodeidentification.mypressonline.com/category/identification-tool/. A similar web-based key was also created for *Pratylenchus* spp. (Nguyen et al., unpublished).

In this study, morphological and molecular characterizations were straightforward; however, molecular data are not available for every plant-parasitic nematode species on GenBank. In the literature, several examples of cryptic species are known, including *Rotylenchus* species (Palomares-Rius et al., 2014). For example, Vovlas et al. (2008) have recognized the subspecies *Rotylenchus magnus jaeni* as a separate (cryptic) species and have proposed the new name *Rotylenchus jaeni*. Cantalapiedra-Navarrete et al. (2013) also described *Rotylenchus paravitis* as a clear example of a cryptic species. Therefore, the combination of morphological and molecular approaches is warranted in order to address the limitations of each approach in the identification of *Rotylenchus* species, or plant-parasitic nematodes in general.

*Rotylenchus rhomboides* n. sp. has relatively large phasmid opening (1.2–2 µm) (see Fig. 1). The size of the phasmid is important as the large phasmid (scutellum) is the only feature to separate *Scutellonema* spp. from *Rotylenchus* spp. According to Germani et al. (1985), *Rotylenchus* species possess pore-like phasmids while *Scutellonema* species have scutella (external opening ca 3–9 µm); although Phillips (1971) described four *Scutellonema* species with a small scutellum (1.2–2.1 µm) in Australia. Germani et al. (1985) transferred these species to the genus *Rotylenchus* after consideration of all morphological and morphometric characteristics, but unfortunately, no molecular data are available for these species. In this study, despite the relatively large phasmids, *Rotylenchus rhomboides* n. sp. is clearly imbedded within the genus *Rotylenchus* according to molecular data.

*Rotylenchus rhomboides* n. sp. was found on banana (*Musa basjoo*) from Japan and *R. buxophilus* was found associated with yam (*Dioscorea tokoro*) from China. Both plants were imported in Belgium but survived for a decade outside under the Belgian weather conditions. Interestingly, *R. buxophilus* has been reported in many countries on many different hosts, including English boxwood (*Buxus sempervirens* var. *suffruticosa* L.), lima beans (*Phaseolus lunatus* L.), rye (*Secale cereale* L.), strawberry (*Fragaria* × *ananassa* Duchesne), tomato, and yew (*Taxus canadensis* Marsh.) in USA; *Cupressus sempervirens* L. and pea in Spain; Hydrangea macrophylla (Thunb.) Ser. in Italy; camellia (*Camellia japonica* L.) in France; unknown grass soils in Austria and Poland; vineyards in Switzerland; bilberries (*Vaccinium myrtillus* L.) in Slovakia; grassland in Romania; bilberry plants in Russia; sugarcane in Taiwan; elder, lilac, and *Taraxacum* F. H. Wigg. in Hungary; beech forest humus in Bulgaria; in Pakistan; olive in Turkey; *Magnolia* sp. L. in Iran; cabbage tree (*Cordyline* sp. Comm, ex R.Br.), *Erica arborea* L., fig *(Ficus carica* L.), and soil from a flower garden in New Zealand (Castillo and Vovlas, 2005). However, *R. buxophilus* has never been reported on yam (*Dioscorea tokoro*). In this study, although the host plant of *R. buxophilus* was imported from China, the above-listed distribution of *R. buxophilus* indicates that this species is more prevalent in Europe compared to Asia, as only one population was recorded from Taiwan.
